# High-fat diet-induced obesity triggers alveolar bone loss and spontaneous periodontal disease in growing mice

**DOI:** 10.1186/s40608-016-0082-8

**Published:** 2016-01-08

**Authors:** Yuko Fujita, Kenshi Maki

**Affiliations:** Division of Developmental Stomatognathic Function Science, Department of Health Promotion, Kyushu Dental University, 2-6-1 Manazuru, Kokurakita-ku, Kitakyushu, 803-8580 Japan

**Keywords:** Obesity, Alveolar bone, Bone architecture, Periodontium, High-fat diet

## Abstract

**Background:**

The relationship between high-fat food consumption and obesity is well-established. However, it is as yet unclear whether high-fat diet (HFD)-induced obesity in childhood and adolescence determines age-related changes in jaw bone health. The aim of this study is to examine the age-related influence of HFD-induced obesity on mandibular bone architecture and the structure of the periodontium in growing mice.

**Methods:**

Male C57BL/6 J mice (6-weeks-old) were divided into two groups (*n* = 6 each): the control group received a control diet and the experimental group a HFD. After treatment for 4, 8, or 12 weeks, trabecular and cortical bone architecture was assessed using micro-computed tomography. The periodontium and alveolar bone structure were evaluated by histopathology.

**Results:**

In HFD mice, body weight, serum total cholesterol, and serum leptin levels were significantly higher than those in age-matched control mice (*p* < 0.05, all comparisons). Reductions in trabecular bone volume and in cortical bone growth (measured as the thickness and cross sectional area) in HFD mice were significant compared with the control mice after 4 weeks of treatment (*p* < 0.05, both comparisons). Significant decreases in cortical bone density in HFD-fed vs. age-matched control mice were determined after 12 weeks (*p* < 0.05). In the HFD mice, the periodontal ligament fibres were disrupted, having lost their orientation with respect to the bone surface, and constriction of the periodontal ligament space was inhibited.

**Conclusions:**

These results suggest that HFD-induced obesity during growth not only triggers mandibular osteoporosis but also increases the risk of spontaneous periodontal disease.

## Background

Trends indicating an increase in the proportion of overweight and obese children and adolescents have been reported in numerous studies from several industrialised countries over the last two decades [1, 2]. Overweight during adolescence continues into adulthood and increases the risk of morbidity from cardiovascular diseases [3]. In addition, adolescents with a below-peak bone mass are at greater risk of developing osteoporotic fractures when they reach old age.

The development of obesity and osteoporosis in adults can be traced to their dietary intake and physical activity during childhood and adolescence. While, the relationship between high-fat food consumption and obesity is well-established [4], whether it affects bone health remains controversial.

Animal studies have shown a positive correlation between obesity and bone density [5] whereas, at least in mice, high-fat diet (HFD)-induced obesity correlates negatively with bone mass [6, 7].

Several studies in rats have also linked obesity with type 2 diabetes, and hypercaloric diet induced-obesity with morbidity from periodontal diseases [8, 9]. A HFD also contributes to obesity in association with a state of chronic inflammation, both in animals [10, 11] and humans [12]. However, it is as yet unclear whether HFD-induced obesity in childhood and adolescence determines age-related changes in alveolar bone architecture and oral bone health.

In the present study, we evaluated the influence of HFD-induced obesity on alveolar bone quantity and quality, using micro-computed tomography (micro-CT), and performed a histopathological evaluation of the mandible of growing mice to determine potential adverse effects of obesity on oral bone health.

## Methods

### Animal care

Male C57BL/6 J mice (6 weeks old) were purchased from Charles River Japan (Kanagawa, Japan). The animals were housed individually under a 12/12-h light/dark cycle at a constant temperature of 22 ± 1 °C and a humidity of 50 ± 5 %. After acclimation for 1 week on a standard pellet chow, in which 16.4 % of total calories were obtained from lipids (AIN-93G) [13], the mice were divided randomly into seven groups (*n* = 6 each). The mice in one group were euthanized immediately with pentobarbital sodium at 7 weeks of age. Three groups remained on the standard diet (control groups), and the other three groups were switched to a high-fat chow (62 % of energy from fat; HFD groups). Both diets were purchased from the Oriental Yeast Co. (Tokyo, Japan; Table 1). Food intake, expressed as the food weight consumed (g) per mouse per day, was assessed by weighing the food in each cage dispenser, including the food that was spilled on the floor of the cage, and normalised among the six groups. The mice had access to tap water *ad libitum* throughout the study. Body weight was recorded weekly. After 4, 8 and 12 weeks, mice in both the control and HFD groups were euthanized using pentobarbital sodium. Their right and left hemi-mandibles were harvested bilaterally, cleaned of adherent tissue and used for micro-CT analysis and for histopathological evaluation, respectively. All animal procedures were approved by the Committee for Care and Use of Laboratory Animals of Kyushu Dental University (08-012).

**Table 1:** Composition of experimental diet

	Standard	High-fat
	w/w (%)	
Milk casein	20.0	25.6
L-cystine	0.3	3.6
maltodextrin	0	6.0
Corn starch	39.7486	0
α-Corn starch	13.2	16.0
Sucrose	10.0	5.0
Soy bean oil	7.0	2.0
Lard	0	33.0
Cellulose powder	5.0	6.61
Mineral mix (AIN-93G)	3.5	3.5
Calcium carbonate	0	0.18
Vitamin mix (AIN-93G)	1.0	1.0
Choline bitartrate	0.25	0.25
The third butyl hydroquinone	0.0014	0
Calorie (kcal/100 g)	377.0	506.2
Ratio of fat/total calorie (%)	16.4	62.2

### Serum analyses

Blood was obtained from mice under anaesthesia. Serum was isolated by centrifugation (3000 rpm, 15 min) and frozen at -80 °C. Serum total cholesterol, low-density lipoprotein (LDL), high-density lipoprotein (HDL) and triglycerides levels were quantified using routine laboratory methods (Nagahama Life Science Laboratory, Shiga, Japan). Serum insulin and leptin levels were measured using enzyme-linked immunosorbent assay (ELISA) kits for mouse insulin (Ohtsuka, Ltd., Tokyo, Japan) and mouse leptin (Morinaga Institute of Biological Science, Inc., Tokyo, Japan) according to the manufacturers’ protocols. The precision of the ELISAs was determined as the coefficient of variation (CV, %) and expressed as a percentage. The intra-assay CV ranged from 3.8 to 15.7 % and the inter-assay CV from 5.2 to 15.1 %.

### Micro-computed tomography (micro-CT)

**Fig. 1:**
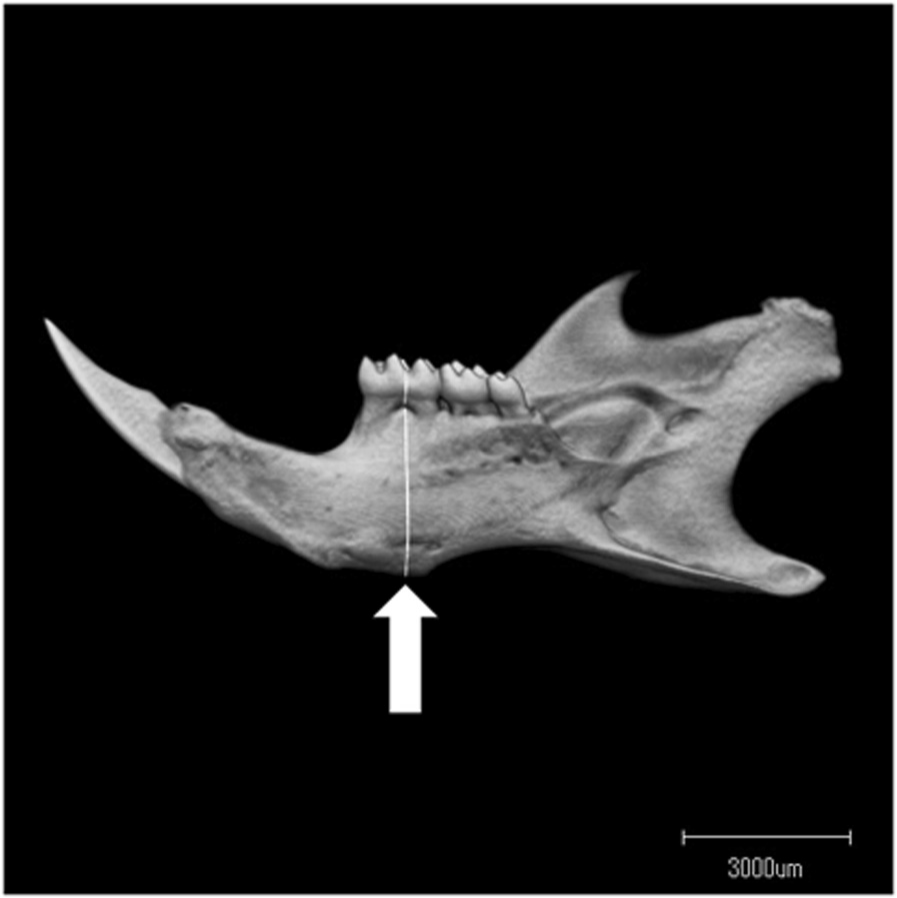
Lingual view of the mandible as seen on micro-CT. The morphological analysis focused on the furcation zone between the mesial and distal roots of the mandibular first molar region (white line and arrow)

The Scan Xmate-L090 (Comscantecno Co., Kanagawa, Japan) micro-CT instrument was used to image representative mandibles from control and HFD mice. The selected measurement site was as a buccal-lingual cross-slice of the first mandibular molar in the furcation zone (between the mesial and distal roots), as described previously [14] (Fig. 1). To distinguish cortical bone, trabecular bone and bone marrow in the analysis, the adaptive threshold was adjusted in the three-dimensional image-analysis software TRI/3D-BON; Ratoc System Engineering, Tokyo, Japan) according to the manufacturer’s instructions. The samples were scanned at a 10-μm resolution, and tissue volume (TV, mm^3^) and trabecular bone volume (BV, mm^3^) were measured directly. The trabecular bone volume fraction (BV/TV, %), trabecular number (Tb.N, 1/mm), trabecular thickness (Tb.Th, μm) and trabecular separation (Tb.Sp, μm) were calculated. Cortical bone volume (Cv, mm^3^), the void volume of cortical bone (Vv, mm^3^), cortical bone thickness (Ct.Th, μm), external bone length (Ex.L, μm) and endosteal bone length (En.L, μm) were measured directly. The cortical bone density (Ct.BD: 1-Vv/Cv, %) and cortical bone cross sectional area (Ct.CSA, mm^2^) were also calculated [15].

### Histological analysis

Left mandibles isolated from euthanized mice were fixed in 10 % neutral-buffered formalin, decalcified for 2 weeks with 10 % EDTA (pH 7.4), dehydrated with increasing concentrations of ethanol, and embedded in paraffin wax. Sections were cut from the paraffin wax blocks at a thickness of 4 μm and stained with haematoxylin and eosin. Images were taken using the Eclipse E200 microscope camera system (Nikon. Tokyo, Japan).

The periodontium and alveolar cortical bone were imaged at the mid- sections, mesiodistally of the mesial root of the mandibular first molar [14].

### Statistical analysis

All data are expressed as medians and interquartile ranges of six animals per group. Comparisons between diet (HFD *vs*. standard chow) and among various time points (11, 15 and 19 weeks vs. 7 weeks) were performed using the by Mann–Whitney *U* test. A *p* value less than 0.05 was considered to indicate statistical significance. Statistical analyses were conducted using SPSS version 23.0 for Windows (IBM Japan, Inc., Tokyo, Japan).

## Results

### Body weight and serum parameters

**Fig. 2:**
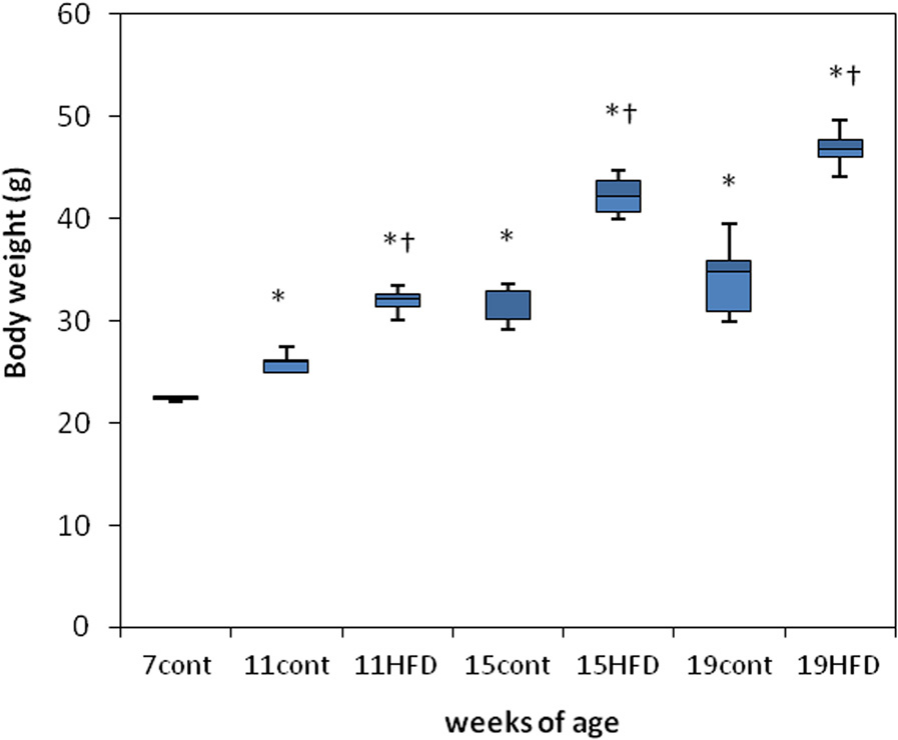
Body weights in mice fed a standard diet (cont) or a high-fat diet (HFD). The data are displayed as a box-and-whisker plot. The boxes indicate the intervals between the 25^th^ and 75^th^ percentiles, and the horizontal bars inside the boxes indicate the medians. The whiskers indicate the data intervals. ^*^ *P* < 0.05, vs. 7-week-old mice and ^†^ *P* < 0.05, vs. age-matched control mice

**Fig. 3:**
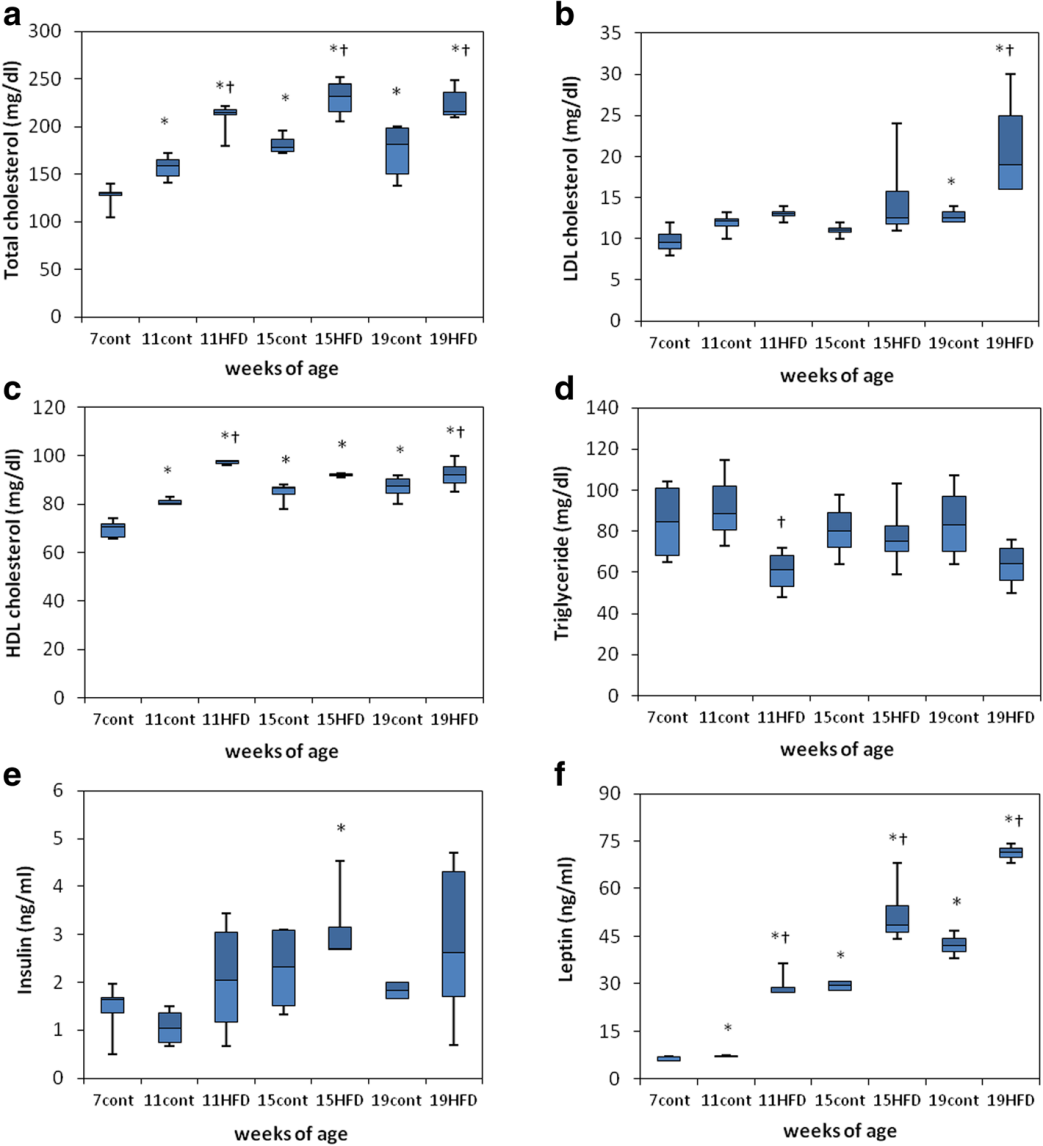
Serum parameters in mice fed a standard diet (cont) or a high-fat diet (HFD). Total cholesterol (**a**), LDL cholesterol (**b**), HDL cholesterol (**c**), triglyceride (**d**), insulin (**e**), and leptin (**f**). The data are displayed as a box-and-whisker plot. The boxes indicate the intervals between the 25^th^ and 75^th^ percentiles, and the horizontal bars inside the boxes indicate the medians. The whiskers indicate the data intervals. ^*^ *P* < 0.05, vs. 7-week-old mice and ^†^ *P* < 0.05, vs. age-matched control mice

Body weight increased significantly in the HFD group compared with the age-matched controls at 11, 15 and 19 weeks (Fig. 2), as did total cholesterol and leptin levels (Fig. 3a and f). Serum LDL cholesterol levels were significantly higher in the HFD group than in the age-matched controls at 19 weeks (Fig. 3b), and serum HDL levels were higher at 11 and 19 weeks (Fig. 3c). Serum triglyceride levels were significantly lower in the HFD group than in the age-matched controls at 11 weeks (Fig. 3d). Serum leptin levels increased with age in the control group (Fig. 3f).

### Quantitative evaluation of alveolar bone architecture

**Fig. 4:**
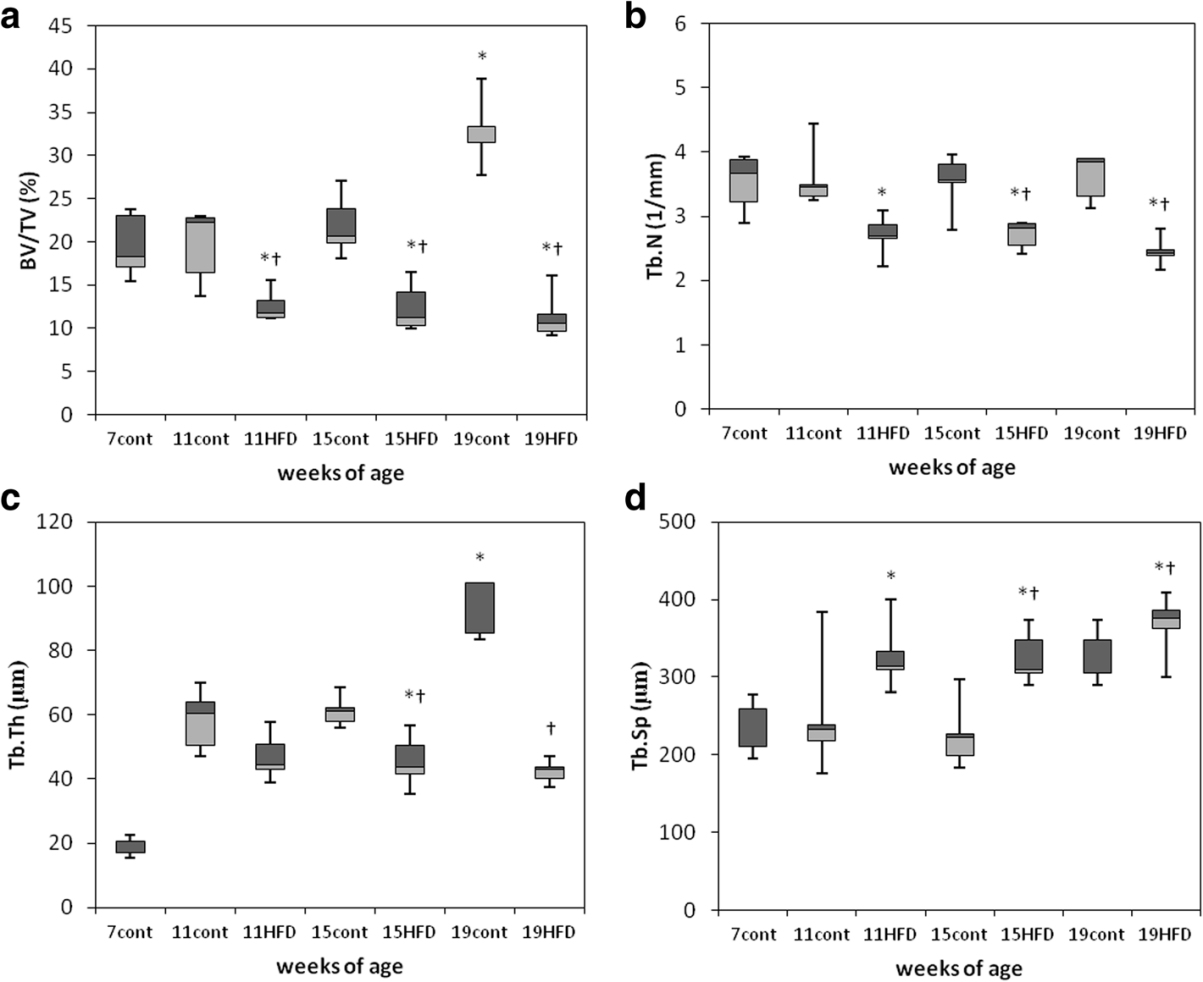
Micro-CT analysis of trabecular bone in the mandible. Bone architectural parameters of trabecular bone volume fraction (BV/TV, **a**), trabecular thickness (Tb.N, **b**), trabecular number (Tb.Th, **c**) and trabecular separation (Tb.Sp, **d**) of the furcation zone between the mesial and distal roots of the mandibular first molar in mice fed a standard diet (cont) or a high-fat diet (HFD). The data are displayed as a box-and-whisker plot. The boxes indicate the intervals between the 25^th^ and 75^th^ percentiles, and the horizontal bars inside the boxes indicate the medians. The whiskers indicate the data intervals. ^*^ *P* < 0.05, vs. 7-week-old mice and ^†^ *P* < 0.05, vs. age-matched control mice

The BV/TV of the trabecular bone of 11-, 15-, and 19-week-old mice was significantly lower in the HFD group than in the age-matched controls (Fig. 4a). In 15- and 19-week old mice Tb.N and Tb.Th were significantly lower in the HFD group (Fig. 4b and c) and Tb.Sp significantly higher (Fig. 4d).

**Fig. 5:**
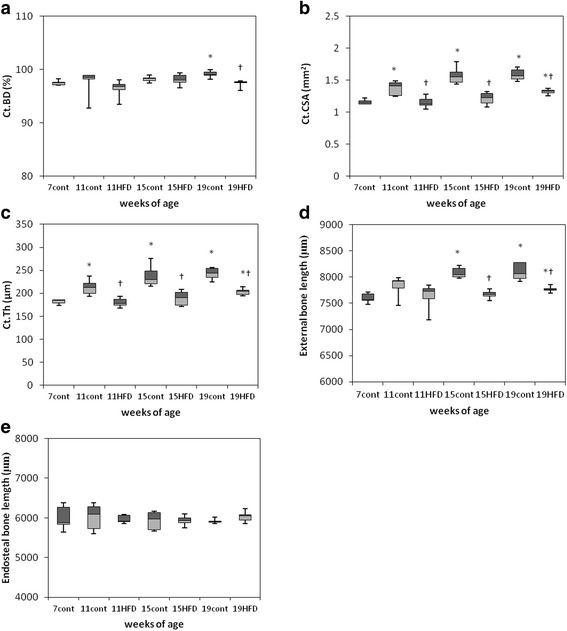
Micro-CT analysis of cortical bone in the mandible. Bone architectural parameters of cortical bone density (Ct.BD, **a**), cortical bone cross sectional area (Ct.CSA, **b**), cortical bone thickness (Ct.Th, **c**), external bone length (**d**), and endosteal bone length (**e**) of the furcation zone between the mesial and distal roots of the mandibular first molar in mice fed a standard diet (cont) and a high-fat diet (HFD). The data are displayed as a box-and-whisker plot. The boxes indicate the intervals between the 25^th^ and 75^th^ percentiles, and the horizontal bars inside the boxes indicate the medians. The whiskers indicate the data intervals. ^*^ *P* < 0.05, vs. 7-week-old mice and ^†^ *P* < 0.05, vs. age-matched control mice

In the cortical bone, Ct.BD was significantly lower in the HFD than control group at 19 weeks (Fig. 5a), whereas Ct.CSA and Ct.Th were significantly lower in the HFD group at all three time points. However, age-dependent increases in Ct.CSA and Ct.Th were recorded over the course of the experiment and showed significantly higher values in 19-week-old mice (Fig. 5b and c). Compared with the control group, Ex.L was significantly lower in 15- and 19-week-old HFD mice; however, when compared with the values at the beginning of the experiment, Ex.L was significantly higher in 19-week-old mice (Fig. 5d).

### Micro-CT imaging of alveolar bone architecture

**Fig. 6:**
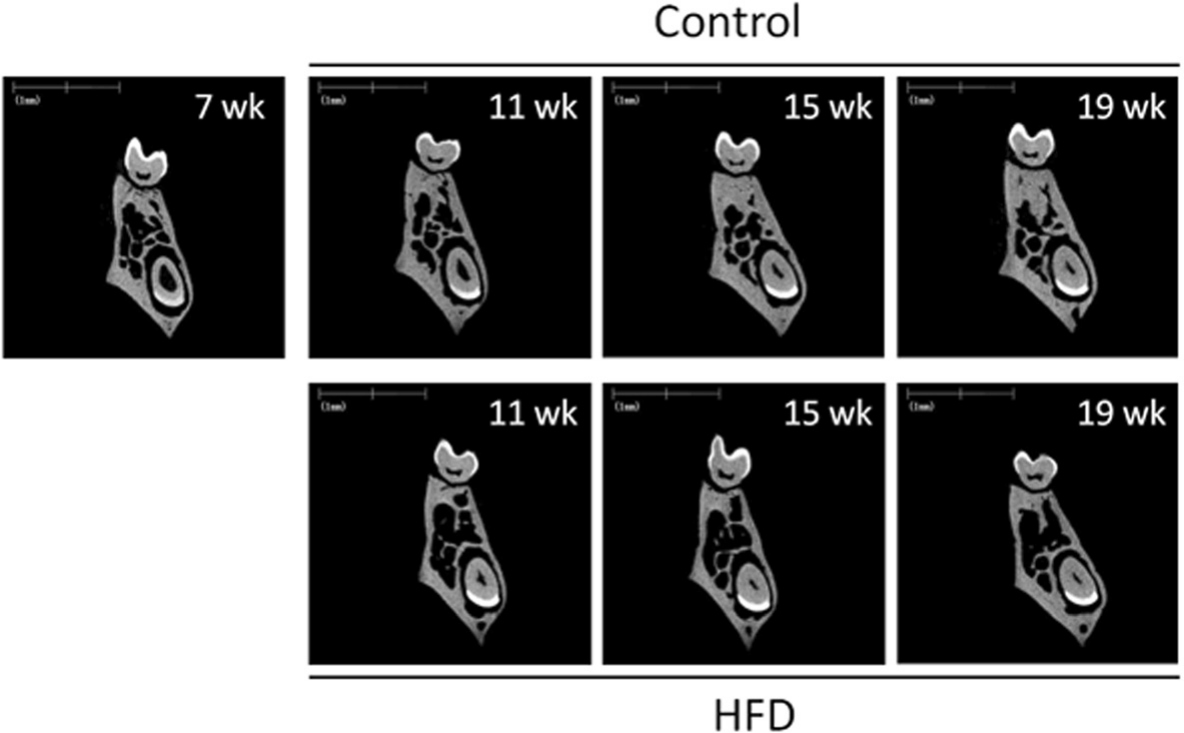
Representative micro-CT cross-sectional transverse scans of the furcation zone of the mandibular first molar in mice fed a standard diet (Control) or a high-fat diet (HFD)

Representative micro-CT scans of the mandibles from HFD-fed vs. control mice showed clear morphological abnormalities in the former, including age-dependent increases in bone marrow cavities and decreased trabecular connectivity (Fig. 6).

### Histological evaluation of periodontium

**Fig. 7:**
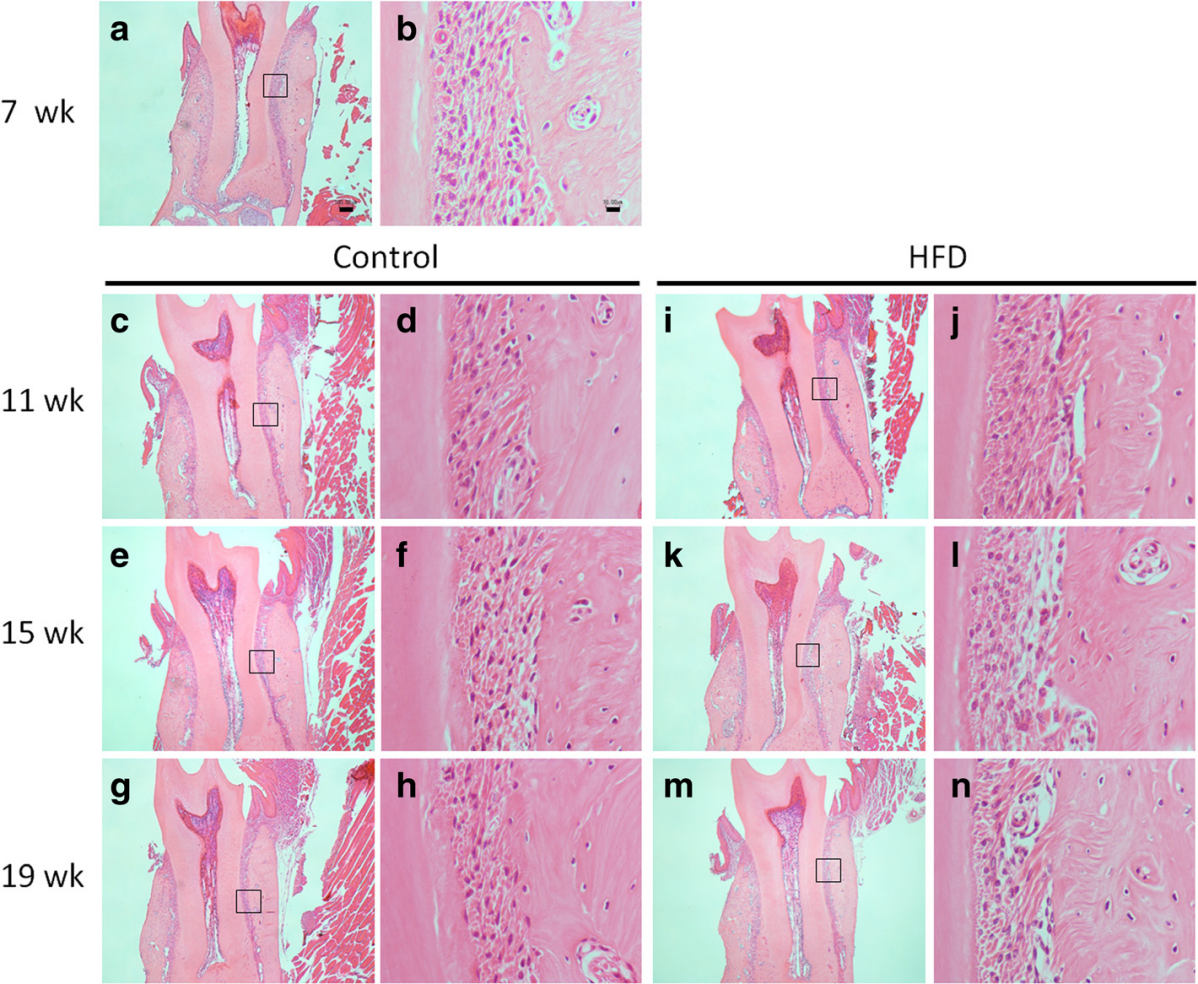
Histological images of the PDL of the mesial root of the mandibular first molar in mice fed a standard diet (Control) or a high-fat diet (HFD). A higher magnification of the boxed regions in (**a, c, e, g, i, k** and **m**) is shown in (**b, d, f, h, j, l** and **n**), respectively. Sections are stained with hematoxylin and eosin. Original magnification **a, c, e, g, i, k** and **m**: ×40. Original magnification **b, d, f, h, j, l** and **n**: ×100. Bar a = 100 μm. Bar b = 10 μm

The results of the histological evaluation of the buccal cortical bone, using samples obtained from the middle mesiodistal area of the mesial root of the first molar, are presented in Fig. 7. In the control group at 11, 15 and 19 weeks, the cortical lamellar bone was well-organised, and the periodontal ligament (PDL) fibres were oriented obliquely. The PDL spaces exhibited age-dependent narrowing in the control group (Fig. 7c-h).

Evidence of alveolar bone resorption in the HFD group included increased numbers of osteoclasts on the bone surface of the PDL spaces of 15-week-old mice. In these mice, disruption of the PDL fibres, accompanied by inflammatory cell infiltration, was seen on the surface of the alveolar bone (Fig. 7l). Disruption of the PDL fibres was also apparent on the bone surface of 19-week-old HFD-fed mice, accompanied by pronounced vasodilatation (Fig. 7n). The PDL spaces were at all time points in HFD mice than in the age-matched control mice (Fig. 7i-n).

## Discussion

Significant increases in body weight and serum cholesterol levels, together with significant decreases in bone quantity and quality, were found in HFD-induced obese mice. In the trabecular bone of these mice, deterioration of the trabecular bone architecture resulted in an overall decrease in mandibular BV/TV, as determined by micro-CT. Although cortical bone formation was slower in HFD-fed than in control mice, bone formation on the periosteal surface increased with age in both groups. Additionally, bone resorption on the endosteal surface was slightly higher in HFD-fed mice than controls, as seen on micro-CT. The HFD-fed mice at 19 weeks also had a significantly lower in Ct.BD than that of the controls, consistent with a significant increase in the porosity of cortical bone at the end of the experiment.

This study demonstrated a significant increase in serum leptin levels in HFD-fed mice compared with their age-matched controls, although the levels also increased in the latter.

Leptin is known as an important circulating signal that inhibits food intake and enhances energy expenditure through its actions in the brain [16]. However, several studies have shown that a HFD plays key role in the development of leptin resistance in animals [17, 18]. In another study, energy expenditures were lower in mice fed a high-fat versus a low-fat diet, even though intake was similar between two groups [19]. Energy expenditure inhibition due to leptin resistance leads to abnormal accumulation of triglycerides in the liver and other organs, since ingested triglycerides are not being used as an energy source.

We found that serum triglyceride levels tended to be lower and serum HDL cholesterol levels higher in HFD-fed mice than in control mice. These findings are in contrast to those of previous studies [20, 21] but are consistent with those of Graham et al. [22]. The differences may be related to whether serum leptin levels exceed the capacity of the body’s transport system, including the entry of leptin into the cerebrospinal fluid. Additionally, in HFD-fed mice, an increase in serum total cholesterol levels is probably followed by an increase in serum HDL cholesterol levels.

The causes of leptin resistance are unclear, but hyper-nutrition leads to endoplasmic reticulum stress, and thus to inflammation, in adipose tissue [23–25]. Oxidative stress was also shown to induce leptin resistance in HFD-fed mice [26]. The inflammation in HFD-fed mice is accompanied by increased expression of inflammatory cytokines, such as interleukin (IL)-6, IL-1β, and tumor necrosis factor-α, in adipocytes and macrophages via activation of the c-Jun N-terminal kinase and nuclear factor-κB pathways [24, 26]. Recently, Dib et al. reported that leptin acts as a pro-inflammatory adipocytokine in peripheral tissues [27]. These studies suggested that endoplasmic reticulum stress induces inflammation by mediating leptin signals in the adipose tissue of HFD-fed mice.

Other studies have shown a selective increase in the production of reactive oxygen species (ROS) in the adipose tissues of obese mice. These ROS cause both oxidative and endoplasmic reticulum stress, including inflammatory changes in adipose tissue during the course of adipocyte hypertrophy [28]. Moreover, ROS and oxidative stress inhibit osteoblastogenesis [29, 30], suggesting that ROS also inhibit periosteal cortical bone formation in growing HFD-fed growing mice.

The increased expression of inflammatory cytokines and leptin elicit osteoclast activity by regulating the RANKL/RANK/OPG pathway, resulting in increased bone resorption [31, 32].

Thus, in this study, the deterioration of bone structure in the HFD-fed mice could have been due to multiple forms of oxidative stress, which induced leptin resistance and increased inflammation in adipose tissue. However, an age-dependent increase in serum leptin levels within the physiological range did not affect gradual bone growth.

We also showed that HFD-induced obesity during growth increases the risk of mandibular bone osteoporosis and spontaneous periodontal disease. A recent report proposed a link between systemic osteoporosis and periodontal bone loss based on significant up-regulation of inflammatory cytokines in bone and in the bone marrow cells of rats with osteoporosis [33].

In this study, HFD-induced alveolar bone loss may have reflected a state of non-invasive and non-infective inflammation, such as that characteristic of autoimmune disorders. This is in contrast to previous studies that used models of experimental periodontitis [8, 9], as periodontal disease develops as a result of the continuous interaction between host cells and subgingival pathogenic bacteria [34].

The HFD-induced alveolar bone loss in our mice may have been triggered by bacterial endotoxin [lipopolysaccharide (LPS)], a potential inflammatory mediator in mice with HFD-induced obesity [35]. In a recent study, a HFD increased alveolar bone loss in mice injected with LPS [36]. In general, bacterial endotoxins are present in large quantities in the gut [37], and clinical studies have reported the development of postprandial endotoxemia following a high-fat meal [4, 38]. These findings suggest that dietary fats promote the translocation of bacterial endotoxins from the gut into the circulation, where they stimulate periodontal inflammation and alveolar bone loss.

Recently, Suganami et al. proposed the concept of “homeostatic inflammation” in the pathogenesis of non-infectious inflammatory diseases [39]. This may account for the HFD-induced alveolar bone loss in our mice, in which systemic inflammatory changes in bone and other tissues may have developed in association with metabolic stress.

In a previous study, rats fed a high-cholesterol diet showed a modest increase in the distance between the cement-enamel junctions and the alveolar bone crest. The authors suggested that osteoclastic function plays a major role in alveolar bone resorption during increased oxidative stress [40]. However, at a gross histological level, there was no evidence of alveolar bone crest resorption in any of the groups, despite significant increases in serum total cholesterol levels in the HFD-fed mice. These results likely reflected cortical bone formation on the periosteal surface during the growth period. By contrast, the PDL fibres in HFD-fed mice were disrupted, with loss of orientation with respect to the bone surface, and the normal narrowing of the PDL space was inhibited. Narrowing of the periodontium narrows with age, as seen in the control mice, is accompanied by increasing acellular cementum formation and alveolar bone formation. The increased vascular permeability due to inflammatory changes in the blood vessels of the periodontium may promote monocyte adhesion to endothelial cells and migration. In addition, osteoclasts differentiated from those monocytes may have then attached to the alveolar bone surface, resulting in increased alveolar bone resorption in the HFD-fed mice.

Together, these findings suggest that the spontaneous deterioration of periodontal bone is a consequence of HFD-induced obesity during growth.

Two limitations to our study must be noted. First, in the histological evaluation, HFD-fed mice had clear alveolar bone resorption and inflammatory structural changes in the PDL, but systemic bone metabolism was not assessed using serum analyses, such as those measuring bone resorption markers and inflammatory cytokines. Second, bone resorption due to insulin resistance was not considered. Further studies are needed to elucidate the mechanisms of inflammatory alveolar bone resorption induced by a HFD.

## Conclusions

We conclude that the deterioration of trabecular bone architecture and retarded periosteal bone formation begins early in the development of diet-induced obesity and is followed by an increase in cortical bone porosity. We demonstrated that HFD-induced obesity during growth not only triggers mandibular osteoporosis but also increases the risk of spontaneous periodontal disease.

## Abbreviations

BV: trabecular bone volume
BV/TV: trabecular bone volume fraction
Ct.BD: cortical bone density
Ct.CSA: cortical bone cross sectional area
Ct.Th: cortical bone thickness
Cv: cortical bone volume
En.L: endosteal bone length
Ex.L: external bone length
HDL: high-density lipoprotein
HFD: high-fat diet
LDL: low-density lipoprotein
LPS: lipopolysaccharide
micro-CT: micro-computed tomography
PDL: periodontal ligament
Tb.N: trabecular number
Tb.Sp: trabecular separation
Tb.Th: trabecular thickness
TV: tissue volume
Vv: void volume in cortical bone
